# Unusual Presentation of Hypothyroidism in a Pregnant Woman, Mimicking Gestational Trophoblastic Neoplasm

**DOI:** 10.1155/2016/3154267

**Published:** 2016-02-29

**Authors:** Soheila Aminimoghaddam, Narmin Karisani, Maryam Mazloomi, Maryam Rahimi

**Affiliations:** ^1^Department of Obstetrics & Gynecology, Firoozgar Hospital, Iran University of Medical Sciences, Tehran, Iran; ^2^Department of Obstetrics & Gynecology, Shahid Akbar-Abadi Hospital, Iran University of Medical Sciences, Molavi Street, Tehran, Iran; ^3^Department of Obstetrics & Gynecology, School of Medicine, Shahid Akbar-Abadi Hospital, Iran University of Medical Sciences, Molavi Street, Tehran, Iran

## Abstract

Hypothyroidism is a common health issue worldwide with varying clinical manifestations. We report a woman who experienced an incomplete abortion and undiagnosed hypothyroidism who was referred to the oncologist with the suspicion of metastatic gestational trophoblastic neoplasm (GTN). A 29-year-old woman with incomplete abortion was referred to an oncologist for possible GTN due to persistent active vaginal bleeding, an elevated beta human chorionic gonadotropin (hCG), abnormal cervical inspection exam, abnormal liver function tests, ovarian enlargement, ascites, and a pleural effusion. She was found to have hypothyroidism in further work-up. She was managed with thyroid hormone replacement therapy and her condition improved after 6 weeks. Complete resolution of the ovarian mass and pericardial and pleural effusion was achieved. This case describes an important experience; hypothyroidism should be considered in the differential diagnosis of any woman with an incomplete abortion presenting with an ovarian mass. Evaluation and correct diagnosis are important to prevent mismanagement.

## 1. Introduction

Evaluation of thyroid disease in pregnancy is important for gestational maternal health, obstetric outcome, and subsequent development of the child. The most frequent thyroid disorder in pregnancy is hypothyroidism. Maternal hypothyroidism increases the risk for miscarriage and fetal death [1], anemia, postpartum hemorrhage, placental abruption, cardiac dysfunction [2], gestational hypertension/preeclampsia, and preterm births [1].

Gestational trophoblastic diseases (GTD) are rare complications of pregnancy caused by defective differentiation of the trophoblast. Symptoms differ and may range from uterine bleeding to metabolic disease such as human chorionic gonadotropin (hCG) triggered hyperthyroidism. Metastasis to the lungs and vagina is possible. Additionally, lutein-cysts of the ovaries can occur as a consequence of increased *β*-hCG resulting in ovarian hyperstimulation [3].

Ovarian hyperstimulation syndrome (OHSS) not only happens mostly as an iatrogenic complication of assisted reproductive technology but also occurs following high levels of hCG and severe hypothyroidism [4, 5]. Spontaneous and iatrogenic OHSS share similar pathophysiological sequences including massive recruitment and growth of ovarian follicles, extensive luteinization provoked by hCG, and oversecretion of vasogenic molecules by the corpus luteum. This can cause acute fluid shifts from the intravascular space to third-space compartments [6, 7]. Due to differences in the treatment of these different diseases, a correct diagnosis is essential [3, 4, 8].

We report a case of hypothyroidism that was initially misdiagnosed and referred to an oncologist with suspicion for metastatic gestational trophoblastic neoplasm. The history and all laboratory tests must be interpreted with care because misinterpretation can lead to serious consequences for the patient and a delay in treatment.

## 2. Case Presentation

**Figure 1:**
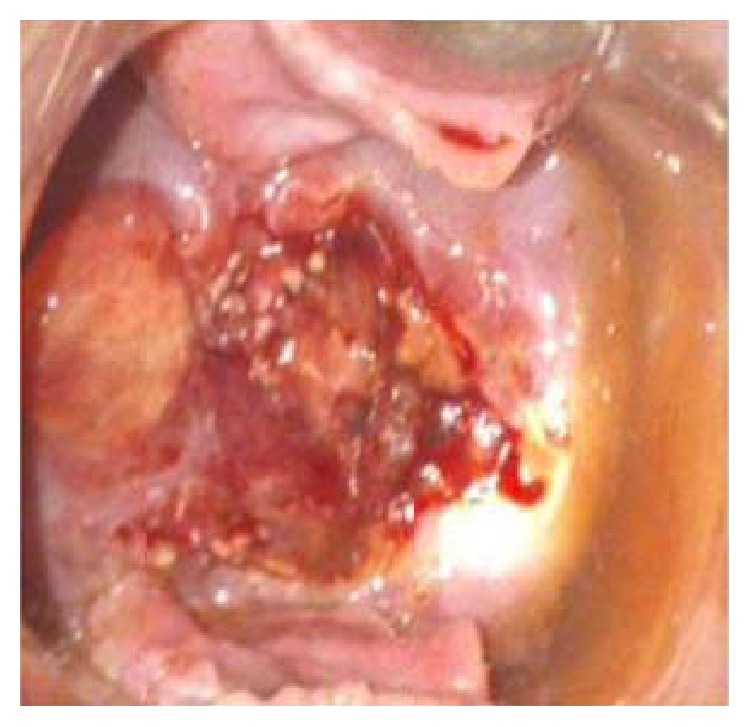
Abnormal cervical inspection.

A 29-year-old woman presented at 13 weeks of gestational age to Robat Karim Hospital in a poor suburb of Tehran, Iran, with abdominal pain and severe vaginal bleeding. She complained of having spontaneously expelled some tissue at home. Upon examination, her cervical os was noted to be open with active, severe vaginal bleeding and retained products of conception inside. She was very pale with a pulse rate of 110 bpm, blood pressure of 100/60 mmHg, body temperature of 36.2°C, and respiration rate of 26/min. Her hemoglobin level was 7.6 g/dL. She was resuscitated and given syntometrine, and she was advised to undergo a dilatation and evacuation (D and E), procedure due to retained products of conception. The patient was noted to have persistent active bleeding after 2 days and the level of beta human chorionic gonadotropin was 50,000 mUI/mL. Abdominal and transvaginal ultrasonography showed a heterogeneous mass with increased vascularity in the uterus with bilateral ovarian multilocular masses, and a large amount of ascites. She was referred to our department on suspicion of metastatic GTN. Physical examination of our patient on admission revealed normal vital signs and a severely distended abdomen with evidence of ascites. Vaginal examination showed slight bleeding and cervical changes (Figure 1). The patient underwent cervical biopsies for pathological diagnosis.

**Figure 2:**
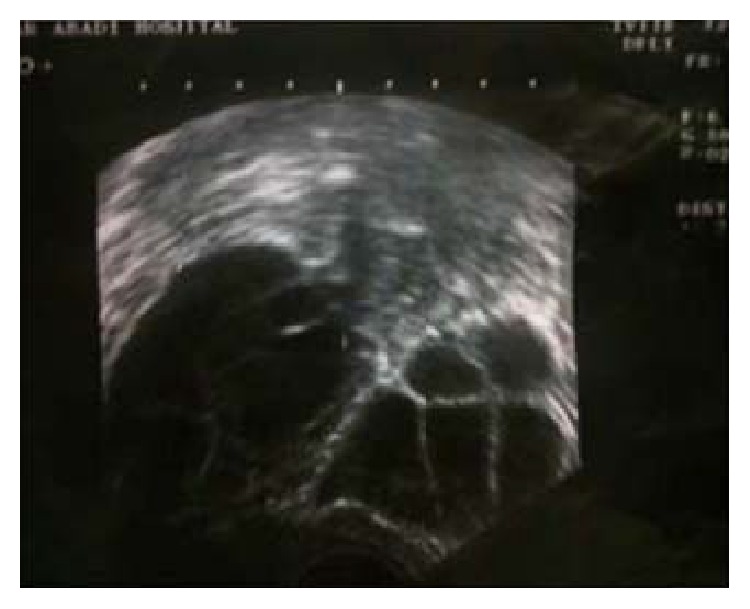
Ultrasound image shows bilateral ovarian multilocular cysts extending to the midabdomen.

Her medical history revealed hypothyroidism that was under treatment for 3 years but she had stopped her medication on her own. Initial laboratory tests showed that Hb was 8.5 g/dL; blood electrolytes, creatinine and blood urea nitrogen, and coagulation profile were normal but liver enzymes were elevated (AST = 113 U/m, ALT = 67 U/m). Beta human chorionic gonadotropin was 30,000. The tumor markers included CA-125 (<1000 IU/mL), and CA 19-9 and CEA were reported normal. Abdominal and transvaginal ultrasonography showed a heterogeneous endometrial lining in the uterus, with mild ascites and bilateral ovarian multilocular cysts extending to the midabdomen (Figure 2). A right pleural effusion was also present on chest X-ray. Abdominal pelvic scan showed enlarged multicystic ovaries (7 cm right, 12 cm left) with ascites. Chest spiral computed tomography (CT) scan showed pericardial and right pleural effusion. Brain MRI was normal. Regarding the medical history, TSH was >100 mIU/L, T_4_ = 2.8 *μ*g/dL (normal: 4.5–12.5 *μ*g/dL), and T_3_ = 0.8 *μ*g/dL (1.18–3.4). The serum *β*-hCG showed regression (3200→1705→599→100). Endocrinology consultation recommended levothyroxine 100 *μ*g/day. Other laboratory tests were as follows: LDL = 130 mg/dL, TG = 380 mg/dL, and Chol = 200 mg/dL. Curettage was done at Robat Karim Hospital and the results showed products of conception with no molar pregnancy and no malignancy. Cervical biopsy reported inflammation and decidualized reactions. According to the good general condition and discontinuation of vaginal bleeding, she was discharged. On close follow-up, imaging studies revealed reduced pleural and pericardial effusion. Bilateral ovarian cysts became significantly smaller 6 weeks after levothyroxine replacement, and vaginal inspection was normal.

In our center, two important inconsistencies with the original assessment were evaluated. First, a thorough medical history highlighted that the patient had hypothyroidism but stopped taking levothyroxine three years ago. Secondly, inadequate lab tests were requested. These findings significantly reduced the likelihood of metastatic gestational trophoblastic neoplasm (GTN) and confirmed the diagnosis of OHSS in hypothyroidism.

## 3. Discussion

This case highlights a diagnosis that is important in patients who are of reproductive age. We have reported a case with hypothyroidism causing ovarian hyperstimulation that was mistaken for metastatic gestational trophoblastic neoplasm (GTN) due to an incomplete approach combined with a reliance on imaging studies that could have potentially led to serious complications. We showed the importance of early diagnosis and the role of observation and early treatment in the resolution of a lethal syndrome.

Gestational trophoblastic disease (GTD) comprises a spectrum of disorders from the premalignant conditions of complete and partial hydatidiform moles through to the malignant invasive mole, choriocarcinoma, and very rare placental site trophoblastic tumor. The malignant forms of the disease are also collectively known as gestational trophoblastic neoplasms (GTN). The most common presenting symptom is vaginal bleeding in the first trimester of pregnancy. Uterine enlargement, preeclampsia, hyperemesis, hyperthyroidism, and respiratory distress are rare [9]. In our case, the history of hypothyroidism, the slow drop in *β*-hCG levels, reported pathological samples based on the lack of evidence of abnormal trophoblastic proliferation or choriocarcinoma, and GTN was unlikely [3, 9].

Hypothyroidism is characterized by a broad clinical spectrum ranging from an overt state of myxedema, end-organ effects, and multisystem failure to an asymptomatic or subclinical condition [10]. Hypothyroidism can cause OHSS. The exact mechanism is not understood clearly and a possible explanation is the preferential formation of estriol in hypothyroid patients. Estriol is a weaker suppressor of gonadotropin release than estradiol and there is excessive gonadotropin release. Another explanation of this rare association is that TSH has weak FSH activity on FSH receptors causing gonadal stimulation [11, 12]. OHSS occurs following high levels of hCG in normal pregnancy, gestational trophoblastic neoplasms, or FSH receptor mutation [7]. Vascular endothelial growth factor (VEGF) is produced in stimulated ovaries, plays a crucial role in the pathophysiology of OHSS, and causes an increase in vascular permeability. Complications from mild OHSS are usually self-limiting. In the more severe forms, fluid shifts can lead to dehydration resulting in acute kidney injury, multiple organ failure, and adult respiratory distress syndrome. Dehydration also increases the risk of thromboembolic phenomena and this occurs in 0.7% to 10% of OHSS patients [4, 13]. In any case, our patient had both hypothyroidism and OHSS complications.

Our patient was not aware of her hypothyroidism symptoms until she became pregnant and developed ovarian hyperstimulation and hypothyroidism complications. Langroudi et al. [14] reported a 15-year-old girl who presented with abdominal pain and distension for a few months. She had classical features of hypothyroidism. She had enlarged ovaries with multiple thin-walled cysts and mild ascites fluid. On follow-up, abdominal ultrasound showed significant reduction of ovary size after 6 weeks of initiation of l-T_4_. Normal ovarian size with complete regression of ovarian cysts was seen after 4 months. But in another study by Sridev and Barathan [15] a pregnant woman with spontaneous conception developed bilateral multiloculated ovarian cystic masses associated with primary hypothyroidism. When treated with levothyroxine, the abdominal discomfort reduced from the 14th week of pregnancy onward and the size of ovaries normalized at 20 weeks. Strafford et al. [16] presented a case of ovarian hyperstimulation syndrome occurring after evacuation of a spontaneously conceived hydatidiform molar pregnancy. It showed that ovarian hyperstimulation syndrome may develop in women who undergo treatment for a hydatidiform mole, and serious complications may develop rapidly. Cardoso et al. [17] reported a case of naturally conceived pregnancy associated with spontaneous OHSS and primary hypothyroidism. After increasing the dose of levothyroxine, ovarian size returned to normal at 24 weeks of gestation. Edwards-Silva et al. [5] reported a case of pregnancy with spontaneous OHSS syndrome, uncontrolled hypothyroidism, elevated hCG, deep vein thrombosis, Rh immunization, and enlarged adenexal masses. She was conservatively managed with levothyroxine and heparin; ovarian size was significant regression by 22 weeks of gestation after adequate thyroid repletion. Cesarean delivery of a nonhydropic preterm newborn occurred at 35 weeks of gestation.

This case is an important lesson to all health care providers including gynecologists, surgeons, physicians, and oncologists who should consider hypothyroidism in the differential diagnosis of pregnant woman presenting with multicystic ovarian masses to avoid unnecessary and catastrophic ovarian resection [15].

In pregnant subjects, especially in the first trimester, who present with vaginal bleeding and incomplete abortion, events such as ascites, ovarian enlargement, and pleural effusion may lead to the misdiagnosis of this condition as metastatic trophoblastic disease or other ovarian malignancies and could result in unnecessary exploratory laparotomy or chemotherapy.
